# HOW SPOUSAL BEREAVEMENT SHAPES LIFE SATISFACTION: STABILITY AND CHANGE ACROSS HISTORICAL TIME

**DOI:** 10.1093/geroni/igad104.3400

**Published:** 2023-12-21

**Authors:** Urmimala Ghose, Michael Krämer, David Richter, Gert Wagner, Frank Infurna, Nilam Ram, Denis Gerstorf

**Affiliations:** Humboldt Universität zu Berlin, Berlin, Berlin, Germany; University of Zurich, Zurich, Zurich, Switzerland; SHARE Berlin, Berliln, Berlin, Germany; Max Planck Institute for Human Development (MPIB), Berlin, Berlin, Germany; Arizona State University, Tempe, Arizona, United States; Stanford University, Stanford, California, United States; University of Zurich, Zurich, Zurich, Switzerland

## Abstract

Lifespan psychological and life course sociological research have long shown that spousal bereavement constitutes one of the most stressful life events in older adulthood that is often associated with marked declines in well-being. It is an open question though whether and how the well-being implications of spousal loss have changed over the past decades. To do so, we used multi-year within-person longitudinal change data from 2,042 participants (Mage at event =65.73 years, 71% women) of the German Socio-Economic Panel obtained annually since 1984. We combine latent basis growth models with methods that analyze the Area under the Curve so as to consider the time window from five years prior to five years after spousal loss. Results revealed that having lost one’s spouse in the 2000s and 2010s is associated with overall less well-being declines than having lost one’s spouse in the 1980s and 1990s. Building on earlier approaches that have distinguished anticipation, reaction, and adaptation phases of bereavement revealed that such improvements are primarily driven by fewer declines in the anticipation phase and faster recovery in the adaptation phases (both by about 0.1 SD per 10 years of historical time), whereas we found partial evidence for steeper declines in the immediate reaction phase. We discuss and in part examine possible explanatory factors at the individual, spousal, social, and societal level for the observed historical improvements in life satisfaction trajectories surrounding spousal loss in Germany over the last three decades.

